# An Enhanced Water Solubility and Stability of Anthocyanins in Mulberry Processed with Hot Melt Extrusion

**DOI:** 10.3390/ijms222212377

**Published:** 2021-11-16

**Authors:** Eun-Ji Go, Byeong-Ryeol Ryu, Su-Ji Ryu, Hyun-Bok Kim, Hyun-Tai Lee, Jin-Woo Kwon, Jong-Suep Baek, Jung-Dae Lim

**Affiliations:** 1 Department of Bio-Health Convergence, Kangwon National University, Chuncheon 24341, Korea; a01040363654@daum.net (E.-J.G.); fbqudfuf0419@naver.com (B.-R.R.); 202016297@kangwon.ac.kr (S.-J.R.); 2 National Institute of Agricultural Sciences, RDA, Wanju 55365, Korea; hyunbok@korea.kr; 3 Division of Applied Bioengineering, Dongeui University, Busan 47940, Korea; htlee@deu.ac.kr; 4 Department of Orthopedics, The Catholic University, Seoul 06591, Korea; krnjs99@naver.com; 5 Department of Herbal Medicine Resource, Kangwon National University, Samcheok 25949, Korea

**Keywords:** anthocyanin, alginate, hot-melt extrusion, microcapsule, controlled release

## Abstract

Mulberry fruits are rich sources of anthocyanins that exhibit beneficial biological activity. These anthocyanins become instable in an aqueous media, leading to their low bioavailability. In this study, a colloidal dispersion was produced by processing mulberry samples with hot-melt extrusion. In this process, hydrophilic polymer matrices were used to disperse the compound in an aqueous media. Mulberry samples were processed with hot-melt extrusion and in the presence of an ionization agent and sodium alginate to form mulberry-extrudate solid formulations. The particle size of mulberry-extrudate solid formulations decreased, while the total phenol content, the total anthocyanin content, and solubility increased. Fourier transform infrared spectroscopy (FT-IR) revealed that mulberry-extrudate solid formulations now contained new functional groups, such as –COOH group. We investigated whether mulberry-extrudate solid formulations had a positive impact on the stability of anthocyanins. The non-extrudate mulberry sample and mulberry-extrudate solid formulations were incubated with a simulated gastric fluid system and an intestinal fluid system. The number of released anthocyanins was determined with HPLC. We found that anthocyanins were released rapidly from non-extrudate mulberry extract. Mulberry-extrudate solid formulations contained a large number of available anthocyanins even after being incubated for 180 min in the intestinal fluid system. Thus, hot-melt extrusion enhanced water solubility and stability of anthocyanins with the prolonged release.

## 1. Introduction

Polyphenols are major antioxidants consumed by humans. Polyphenols are widely used in pharmaceuticals, food, and cosmetic industries as natural antioxidants [1].

Anthocyanins (ATCs) are present in high concentrations in fruits, vegetables, and processed foods or beverages, such as juices and wine [2,3]. As the use of synthetic food dyes has raised safety concerns, the demand for ATCs has increased steadily as it is a potential alternative to natural colorants [4].

The advantages of ATCs are as follows: radical scavenging [5], the inhibition of lipoprotein oxidation and the aggregation of platelets [6], anti-inflammatory activity [7], anti-obesity properties [8], improved visual acuity [9], anti-diabetic properties [10], and antioxidant activity [11].

Although ATCs exert antioxidant effects and have numerous advantages, they cause a few problems when used directly by humans. Although many studies have reported on the potential therapeutic effect of ATC in vitro, it has not been clearly reported whether ATC can exert a therapeutic effect in vivo [12]. Furthermore, many studies have reported low bioavailability of ATCs in vivo [13,14].

Researchers have expressed keen interest in analyzing specific physiologically active substances, such as ATC, because they are useful for the promotion of human health. For this purpose, food industries have developed a processing technology to commercialize the use of bioactive substances maintaining function while maximizing bioavailability.

The processing technology has been used for many years to the following reasons: (i) to overcome the sensitivity of the surrounding environment and (ii) to improve the stability of physiologically active substances, which are processed and stored for later use. Among the various processing technologies, encapsulation is implemented in food, nutraceutical industries.

Encapsulation technology protects physiologically active substances from harmful reactions. Moreover, it also prevents their decomposition and extends their shelf life by incorporating chemically active substances into the polymer matrix [15,16,17,18,19]. Apart from protecting the active substances from harsh conditions of a storage environment, encapsulation also efficiently controls the release of bioactive substances at a specific site.

Among the various encapsulation technologies, the hot-melt extrusion (HME) process is suitable for converting bioactive substances, drugs, and materials into products of specific formulation properties, such as a uniform shape and density. Extrusion is to pump raw materials with a proper temperature and pressure [20]. The HME process is used to mix the bioactive substances with a die under controlled conditions [21]. Heat is applied to the material for viscosity control and to allow smooth flow of the food through the die [22,23,24]. Moreover, by changing the physical properties of the solid dispersion, the bioactive substances can be transformed into amorphous or partially amorphous forms: the crystalline structure is broken in the molten system. By converting the crystalline structure into an amorphous structure, researchers can enhance water solubility and the bioavailability of poorly water-soluble substances [25]. In fact, the HME process can also reduce the particle size and increase water solubility by extruding together bioactive substances and polymers [26]. On the other hand, HME possesses some drawbacks. The main disadvantages of HME include the thermal process (drug/polymer stability) and the high flow properties of polymers [27,28]. Therefore, it would be essential to design a proper formulation for thermolabile compounds.

Recently, the HME has been introduced to process natural products. The main purpose is to enhance water solubility and bioavailability of hydrophobic compounds with proper excipients [29,30].

Therefore, the main objective of this study is to develop a colloidal solid dispersion system that enhances water solubility and ensures the controlled release of ATCs in mulberry (MUL). In this study, food-grade polymers, such as alginate were selected for the preparation of colloidal solid dispersion. Thereafter, we determined various physicochemical properties, such as SEM, particle size, and release profile.

## 2. Results and Discussions

### 2.1. Colloidal Solid Dispersion Systems by HME

In order to achieve high water solubility and stability of ATCs in MUL, HME process was introduced with proper excipients (i.e., citric acid and alginate). The addition of acids has been known to enhance stability of ATCs [31]. In addition, alginate could improve water solubility of hydrophobic compounds [32]. HME was introduced to prepare MUL-ESFs. After HME process was done, the extrudate was obtained. For further studies, the extrudates were freeze-dried and milled to achieve the powder form.

### 2.2. An Analysis of Total Flavonoids, Phenolic Content from Different MUL Formulations

The test group’s phenol content was found to be greater than that of the control, but there was only slight difference in the flavonoid content of the samples. MUL is easier to dissolve in ethanol than water, and it was confirmed that the total flavonoid content was high in the ethanol extract. As reported that HME increases the solubility of sample, the total flavonoid content in MUL water extracts treated with HME increased [33,34]. The phenol content was also higher in the ethanol extract than in water, but it was confirmed that it had a positive effect on the total phenol content after HME treatment. This is considered due to the hydrolysis of polyphenols bound to fibers and proteins during the HME process, in addition to increasing the solubility of the HME process, thereby increasing the polyphenol content [35] (Table 1).

### 2.3. An Analysis of ATC Content Obtained from MUL-ESFs

**Figure 1:**
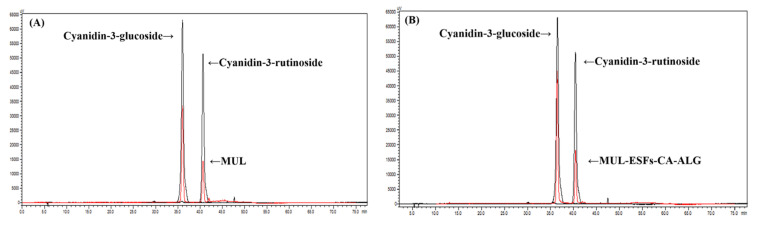
An HPLC chromatogram of cyanidin-3-glucoside and cyanidin-3-rutinoside in MUL. (**A**) MUL, (**B**) solid formulations of the extrudate of MUL treated with 0.5 M citric acid and 0.3% sodium alginate (MUL-ESFs-CA-ALG).

**Figure 2:**
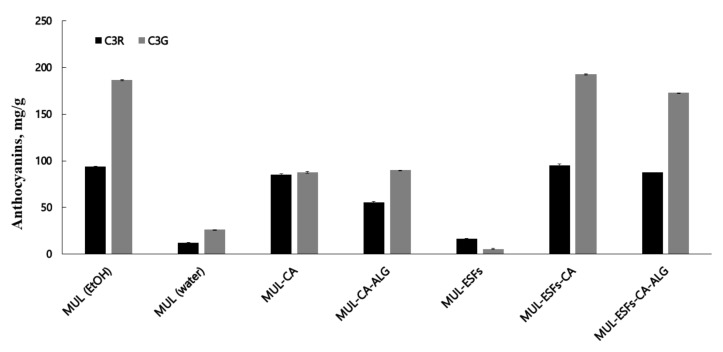
The content of ATCs in MUL extract (mg/g). MUL-CA; MUL treated with 0.5 M citric acid, MUL-CA-ALG; MUL treated with 0.5 M citric acid and 0.3% sodium alginate, MUL-ESFs; only solid formulations of MUL-extrudate, MUL-ESFs-CA; solid formulations of the extrudate of MUL treated with 0.5 M citric acid, MUL-ESFs-CA-ALG; solid formulations of the extrudate of MUL treated with 0.5 M citric acid and 0.3% sodium alginate 0.3%, MUL (EtOH); the extraction of MUL with 80% ethanol, MUL (water); the extraction of MUL with water.

As shown in Figure 1 and Figure 2, the contents of ATCs were higher in extrusion mold than in non-extrusion mold. A strong shear force was applied during the process of extrusion molding. Consequently, the cross-binding and ester bonds of the phenolic compound broke partially within the cell wall. This led to an increase in the extraction efficiency of the phenolic compound [36].

We also found that a* (red) value of extrusion mold was higher than that of non-extraction mold. We compared the a* values numerically with the color difference between samples by using a chromometer. The ATCs were red in color and showed a stable condition in the presence of flavylium cation Compared to the non-extrusion mold, the extrusion mold is dark red in color (Table 2).

### 2.4. Particle Size and Solubility Analysis

**Figure 3:**
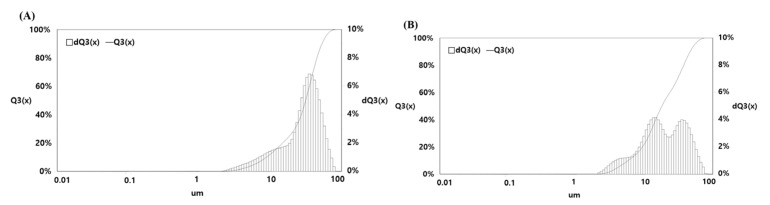
Measuring the particle size distribution of MUL. (**A**) MUL: 29.81 ± 13.80 μm, (**B**) MUL-ESFs-CA-ALG: 23.22 ± 0.26 μm.

As shown in Figure 3, the particle size of the specimen was determined by performing PSA. The particle size was reduced by extrusion molding. By processing the sample by HME, we increased the pressure acting on the mass of the particles and ensured uniform thickness, shape, and size.

Lee [37] stated that HME is the most suitable process for crystalline materials as it reduced the particle size and improved solubility. By reducing the particle size, the surface area of the sample was increased, and active compounds were released. By subjecting MUL samples to extrusion processes, we can unravel the structure of useful ingredients. For this purpose, the particle size of MUL samples was reduced to form a solid dispersant, which further improved solubility and increased bio-utilization.

By measuring the solubility of formulations, we confirmed that solubility of MUL samples improved in extrusion mold. Moreover, solubility was further improved in extrusion mold containing sodium alginate (Table 3). Figure 3 shows the particle size of MUL and MUL-ESFs-CA-ALG. The mean particle size of MUL-ESFs-CA-ALG was smaller than that of MUL. Interestingly, a bimodal population was observed in MUL-ESFs-CA-ALG. This could be explained by the components of natural products like MUL. Natural products contain different components such as fat, carbohydrate, and protein. Among them, fibers are not easy to destroy and degrade by any treatment. MUL formulation treated by HME exhibits two populations, indicating the different structures after the HME process. The particle size has become an important consideration in the field of novel drug delivery systems [38,39]. According to the equation, a smaller particle size equals a larger effective surface area, which increases the rate of dissolution [40] and bioavailability [41]. The melting rate was thus improved by reducing the particle size of MUL samples [42].

### 2.5. FT-IR of Different Formulations

**Figure 4:**
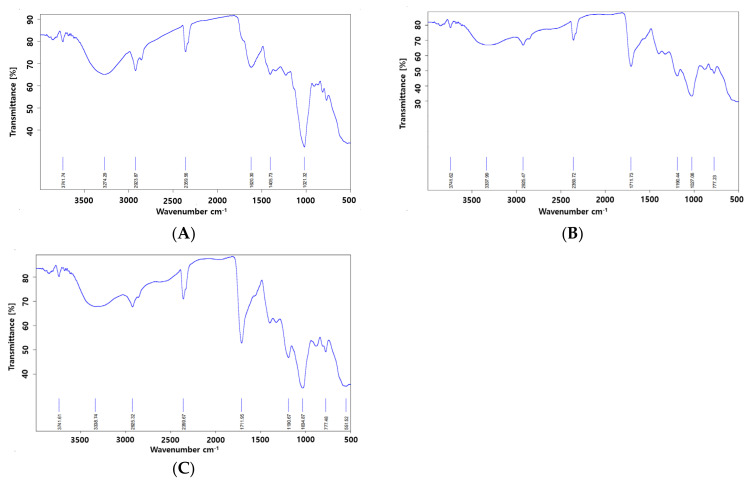
The FT-IR analysis of the MUL extrudate’s solid formulations, which were obtained by different chemical additives. (**A**); MUL powder (MUL), (**B**); solid formulations of the extrudate of MUL treated with 0.5 M citric acid (MUL-ESFs-CA), (**C**); solid formulations of the extrudate of MUL treated with citric acid 0.5 M and 0.3% sodium alginate 0.3% (MUL-ESFs-CA-ALG).

Figure 4 illustrates the FT-IR spectra of different MUL formulations. The peaks observed between 1700 cm^−1^ and 1500 cm^−1^ were attributed to the bending vibrations of methylene and methyl groups. The peaks that appeared between 3500 cm^−1^ and 3000 cm^−1^ were assigned to C–H and –OH groups; these peaks were attributed to the properties of their organic compound. The peaks that appeared at <2000 cm^−1^ represented =C bonds, which were present in carbonyl group compounds in the substituted ring and in aromatic CH bonds [43]. Furthermore, the peaks that appeared below 1500 cm^−1^ were found to be associated with ether, ester, and carbonyl bonds (CO) of carboxylic acid [44].

Figure 4 showed the comparison of the spectrum of non-extrusion sample with that of extrusion sample: the peak that appeared at 1700 cm^−1^ wavelength was a newly produced peak. The peak of 1700 cm^−1^ wavelength indicated the presence of a C=O bond. This indicates that the carboxyl group (COOH) was newly formed by extrusion molding. These results were similar to a previous report that stated the following fact: carboxyl groups are closely associated with the structural changes of compounds, and the appearance of 1700 cm^−1^ wavelength split indicates an increase in the absorption of carboxyl groups [45]. The FT-IR results indicate that HME could be performed on MUL samples containing proper additives to increase the acceptability of the formulation.

### 2.6. The Morphology of MUL Formulations

**Figure 5:**
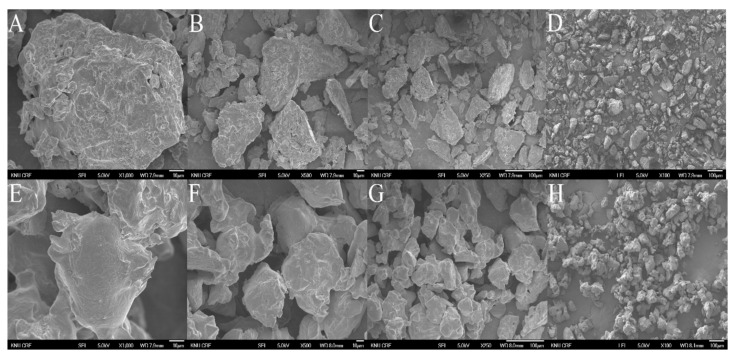
Scanning electron microscopy image of non-extrusion and extrusion. (**A**–**D**) MUL powder (MUL), (×1000, ×500, ×250, ×100), (**E**–**H**) solid formulations of the extrudate of MUL treated with 0.5 M citric acid and 0.3% sodium alginate (MUL-ESFs-CA-ALG), (×1000, ×500, ×250, ×100).

As shown in Figure 5, the morphology of MUL-ESFs-CA-ALG and MUL were visualized with SEM, which was operated at various magnifications (×1000, ×500, ×250, and ×100). The surface of MUL was found to be relatively rough, and the particle size was irregular (Figure 5A–D). In contrast, the surface of MUL-ESFs-CA-ALG was smooth, and the particle size was regular (Figure 5E–H). The uniformity of particle size was associated with increased reproducibility and uniformity in intestinal emission. Previous studies have reported that alginate-coated surfaces reduce the rate of drug release by reducing the smooth pores of water molecules [46].

### 2.7. An In Vitro Release in Different MUL Formulations

**Figure 6:**
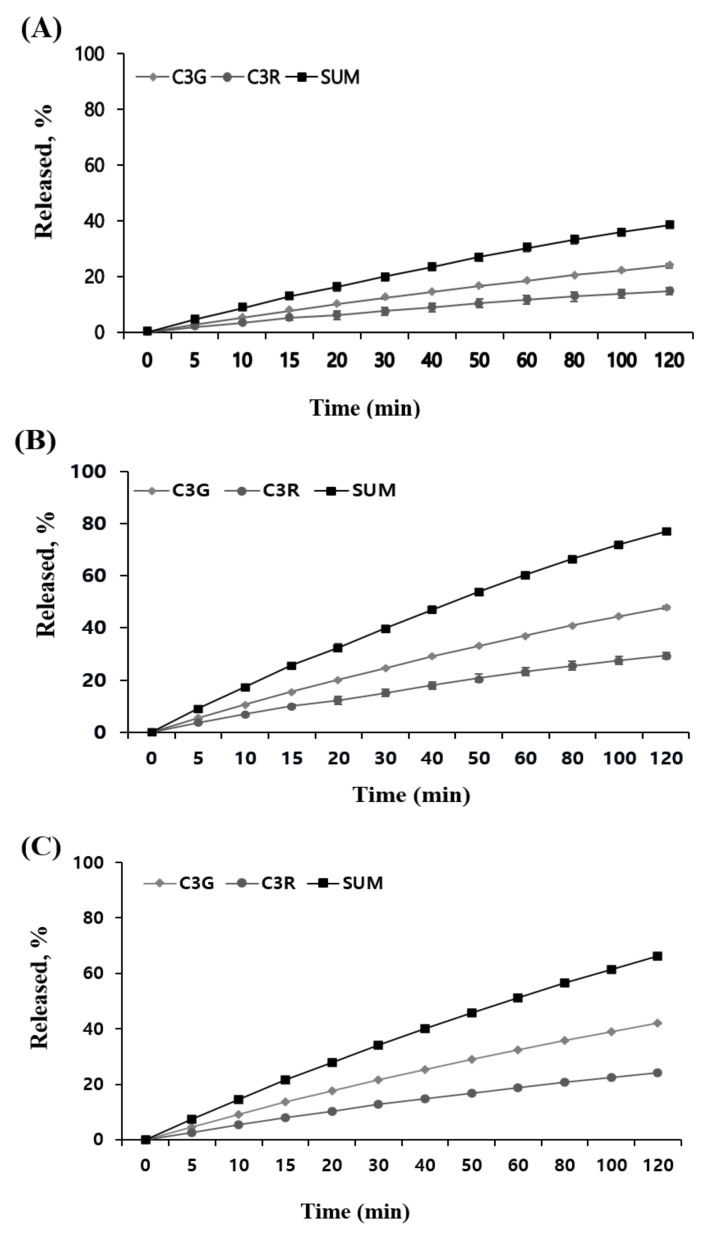
An in vitro release profile of C3G and C3R from (**A**) MUL, (**B**) MUL-ESFs-CA, (**C**) MUL-ESFs-CA-ALG in SGF for 2 h (n = 3, mean ± SD).

**Figure 7:**
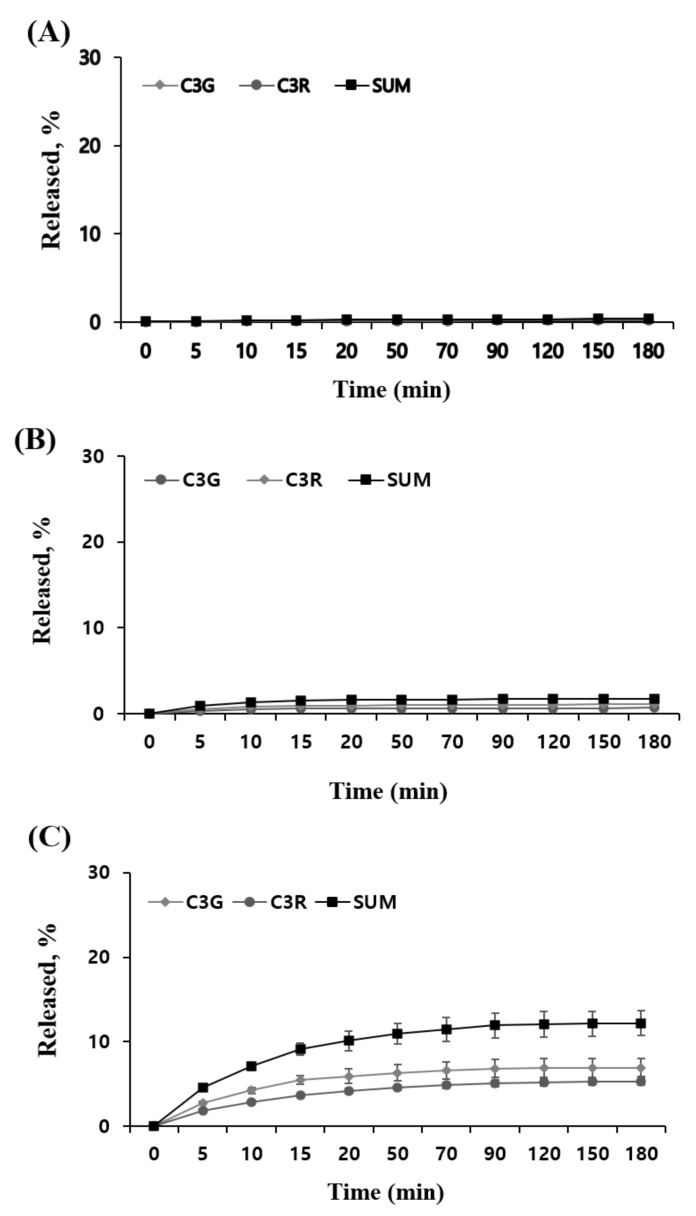
An in vitro release profile of C3G and C3R from (**A**) MUL, (**B**) MUL-ESFs-CA, (**C**) MUL-ESFs-CA-ALG in SIF for 2 h (n = 3, mean ± SD).

Next, the release profile of ATCs in MUL, MUL-ESFs-CA, and MUL-ESFs-CA-ALG samples was determined with SGF and SIF. The study was conducted in an environment that mimics the dissolution of ATCs in SGF and SIF. As shown in Figure 6, two formulations that were prepared by HME exhibited a higher cumulative release rate of two ATCs in SGF as compared to non-processed MUL. This can be explained as follows: firstly, the particle size of formulations was reduced by performing HME process. The increased surface area led to a faster release rate of ATCs from MUL-formulations. Secondly, as discussed earlier, HME processing changed the matrix of processed formulations. The heat and pressure broke down the cell walls in MUL samples. Consequently, more amounts of ATCs were released from formulations. The differences between MUL-ESFs-CA and MUL-ESFs-CA-ALG were attributed to the pH of formulations. We found that the release of ATCs was stable in the flavylium cation, which was found in the gastrointestinal acidic environment (pH 1–3). This finding was similar to a previously reported study that mentions how alginate release rates were stable when the solubility of solid dispersion was reduced [47]. In SIF, we were not able to observe emission characteristics related to MUL (Figure 7). On the other hand, HME-treated formulations exhibited higher cumulative release of ATCs as compared to MUL. This finding is reaffirmed in the emission characteristic that shows how ATCs decompose in small intestine environment (pH 7) [48]. Notably, MUL-ESFs-CA-ALG released a significantly higher amount of ATCs than MUL-ESFs-CA. This result indicates that the diffusion of ATCs is accelerated with the presence of alginate in SIF. In addition, it has been reported that alginate increased surface area and solubility but decreased diffusion [49].

## 3. Materials and Methods

### 3.1. Materials

In this experiment, the chemicals purchased from Sigma-Aldrich (St. Louis, MO, USA) were as follows: caffeic acid, keracyanin chloride, kuromanin chloride, Folin-Ciocalteau reagent, quercetin, pepsin, and pancreatin. Aluminium chloride hexahydrate and potassium acetate were obtained from Junsei Chemical (Tokyo, Japan). Citric acid, sodium alginate, sodium acetate, potassium chloride, and sodium carbonate were purchased from Daejung Chemicals & Metals (Seoul, Korea).

### 3.2. The Preparation of Colloidal Solid Dispersion Systems by HME

**Figure 8:**
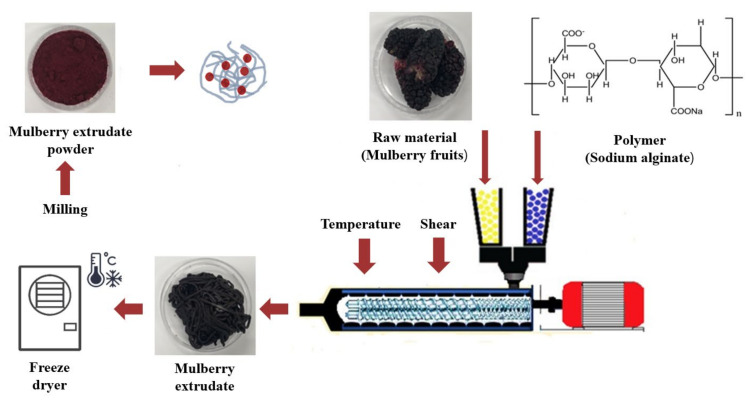
A schematic illustration of mulberry (MUL) production by hot-melt extrusion (HME) process.

In this experiment, raw MUL was processed with HME. In this process, MUL was applied to the copper metal, which was fully loaded onto the biaxial extrusion molder (STS-25HS, co-rotating intermeshing type twin-screw extruder, Pyeongtaek, Korea). The HME process was performed to prepare a solid dispersion of MUL where ATC is encapsulated. The extruder was fitted with a circular die (1 mm). Raw materials were supplied to the extruder at a supply rate of 40 g/min and 150 rpm, and it exerted a high level of shear force with double screws. The temperature profile from the supply area to the die—where the extrusion mold is injected—was as follows: 80 → 100 → 100 → 80 → 70 °C. After the HME process was finished, the extrudate was further dried by a freeze dryer (FDS-5503, Operon Freeze Dryer, Ilshin Biobase, Dongducheon, Korea), followed by pulverization with a mill (HC-BL5100BK_A, Happycall, Gimhae, Korea) to obtain a powder form for further studies. The extrusion mold was manufactured as follows: mulberry powder (MUL), MUL treated with 0.5 M citric acid (MUL-CA), MUL treated with 0.5 M citric acid and 0.3% sodium alginate (MUL-CA-ALG), only solid formulations of MUL-extrudate (MUL-ESFs), solid formulations of MUL extrudates treated with 0.5 M citric acid (MUL-ESFs-CA), and solid formulations of MUL extrudates treated with 0.5 M citric acid and 0.3% sodium alginate (MUL-ESFs-CA-ALG) (Figure 8).

### 3.3. The Total Flavonoid Content Analysis of HME-ESFs

To perform total flavonoid content analysis, we added 0.25 mL of the sample to the test tube. Then, we added 0.75 mL of 95% ethanol, 0.05 mL of 10% aluminum chloride hexahydrate, and 0.05 mL of 1 M potassium acetate. The reaction mixture was allowed to stand at room temperature for 40 min. The absorption of the resultant analyte was measured at 415 nm wavelength by using a UV/VIS spectrophotometer (Genesys 160, Thermo Fisher, Waltham, MA, USA). Quercetin was used as the standard material, and the total flavonoid content of the sample was calculated.

### 3.4. The Total Phenol Content Analysis of Different MUL Formulations

To perform total phenol content analysis, we added 0.05 mL of the sample to the test tube. Then, we added 2 mL of sodium carbonate, 0.05 mL of 50% Folin-Ciocalteu reagent, and 1.4 mL of water. The reaction mixture was allowed to stand at room temperature for 30 min. After the completion of the reaction, its absorption was measured at 750 nm wavelength by using the aforementioned UV/VIS spectrophotometer. Caffeic acid was used as the standard material, and the total phenol content of the sample was calculated.

### 3.5. High Pressure Liquid Chromatography (HPLC) Analysis of Different MUL Formulations

As shown in Table 4, HPLC analysis of the sample was conducted by using 1200 Series systems (Agilent technologies, Waldboronn, Germany). The HPLC column was Triart C18 (5 um, 250 × 4.6 mm, Yamamura Chemical Research Institute (YMC), Kyoto, Japan). The standard material was prepared from kuromanin chloride (C3G) and keracyanin chloride (C3R). The ATC content was determined by calculating the amount of C3G and C3R present in 1 g of MUL.

### 3.6. The Total ATC Content Analysis of Different MUL Formulations

The total ATC content (TAC) was measured by using a differential pH method. The MUL was added into two test tubes that contained two different buffer solutions. Then, a buffer solution of pH 1.0 (0.025 M KCl) was diluted in one test tube by adding a buffer solution of pH 4.5 (0.4 M CH_3_COONa) from another test tube. A ratio of 1:9 (*v*:*v*) was maintained between the buffer solutions and the sample (DF = total volume/sample = 10). After the diluted sample was stabilized for 20 min, its absorbance was measured at wavelengths 510 nm and 700 nm. Distilled water was used as the blank. The absorbance of the sample increased when left for a long time. Therefore, all sample dilutions were measured after being allowed to stand for 20 to 50 min. In addition, the absorbance for each wavelength (220–700 nm) was measured, and the color change was observed at pH 1.0 and pH 4.5 for each sample. The C3G content (TAC, 1) included in the sample was defined as C3G mg present in 1 g of MUL powder.

(1) $$TAC=\frac{A\times MW\times DF\times 1000}{ɛ\times 1}$$

*A* = (A510 nm–A700 nm) pH 1.0—(A510 nm–A700 nm) pH 4.5, *MW* = the molecular weight of cyanidin-3-glucoside, 449.2 g/mol, *DF* = a dilution factor, 1000 = factor for conversion from g to mg, ɛ = the extinction coefficient of cyanidin-3-glucoside, 26,900 ℓ/mol cm, 1 = pathlength in cm

### 3.7. The Chromatic Difference Analysis in Different MUL Formulations

The chromatic difference analysis was performed by using D65-10 light sources (X-rite vs450, X-Rite Inc., Grand Rapids, MI, USA) as the chromatic system. Thus, we compared the luminosity (L*), redness (a*), and yellowness (b*) in CIELC color space.

### 3.8. Particle Characterization

#### 3.8.1. Particle Size Analysis

After pulverizing the sample with the mill, a particle size of 45 μm or less [(using a testing sieve), aperture; 45 μm, wire dia; 32 μm, GhungGeSangSongSa, Seoul, Korea] was subjected to particle size analysis (PSA). The pulverized sample (5 mg) was dissolved in 500 mL water. Then, the particle size of the sample was measured by operating the particle size analyzer (Anaysette-22, Fritsch, Idar-Oberstin, Germany) at a refractive index of 1.38.

#### 3.8.2. Water Solubility

The water solubility of the sample was measured according to a method reported in a previous study [50]. In this process, 50 mL of water was added to 1 g of MUL powder. Then, it was stirred at room temperature for 1 h. The suspension was centrifuged at a speed of 7000× *g* for 15 min. Finally, 10 mL of the supernatant was recovered and freeze-dried at −55 °C.

(2) $$WS\left(\%\right)=\frac{the\;weight\;of\;dried\;supernatant}{the\;weight\;of\;dry\;sample}\times 100$$

#### 3.8.3. Fourier-Transform Infrared Spectroscopy (FT-IR) Analysis

In this study, the molecular structure of the material was analyzed by measuring the absorbance and transmittance of the sample using FT-IR analysis, and information on the formation of new chemical bonds/individuals was confirmed through the results.

#### 3.8.4. Scanning Electron Microscopy (SEM) Analysis

The morphology of samples was photographed by SEM (Zeiss MERLIN Field Emission SEM, Carl Zeiss NTS GmbH, Oberkochen, Germany). Sample was placed on a metal plate and coated with platinum for 60 s after drying. The samples were observed at a voltage of 5 kV.

### 3.9. In Vitro Release Study

#### 3.9.1. Simulated Gastric Fluid (SGF)

To mimic the artificial gastrointestinal environment, 3 g of pepsin was dissolved in 20 mL of water. Then, the suspension was centrifuged at 1500× *g* for 10 min. The resultant supernatant was used as SGF. The MUL powder (0.2 g) was added to 20 mL of citrate buffer (0.1 M, pH 2.0), and then it was mixed for one minute in a shaking water bath (75 ppm, 37 °C). After adding 2 mL of the enzyme solution, 1 mL aliquots were taken at 0, 5, 10, 15, 20, 30, 40, 50, 60, 80, 100 and 120 min. The content of ATCs was determined from the aliquots. In addition, 1 mL of the release medium was replaced with the same volume of fresh medium to maintain the sink condition

#### 3.9.2. Simulated Intestinal Fluid (SIF)

To prepare SIF, 3 g of pancreatin was dissolved in 20 mL of water. Then, it was centrifuged at 1500× *g* for 10 min, and subsequently, the supernatant was used as SIF. The MUL powder was added to 20 mL phosphate buffer (pH 7.0) and mixed for one minute in a shaking water bath (75 rpm, 37 °C). After adding 2 mL of the enzyme solution to this reaction mixture, 1 mL aliquots were taken every 0, 5, 10, 15, 20, 50, 70, 90, 120, 150, 180 min to analyze the content of ATCs. Moreover, 1 mL of the release medium was replaced with the same volume of fresh medium to maintain the sink condition.

### 3.10. Statistical Analysis

The results of this work were expressed as means, and the statistical significance was determined by SPSS software (IBM, Armonk, NY, USA). The SPSS software also analyzed the difference between each experimental group by performing one-way ANOVA at the *p* < 0.05 level, and the results were verified by the Turkey’s Range Test.

## 4. Conclusions

In conclusion, ours is a novel study that proves it is feasible to treat MUL samples with HME. The results confirmed that HME processing and the addition of citric acid and sodium alginate resulted in the decomposition of the chain of alginate polymers, causing a high concentration of released ATCs in the molecular structure of the polymer matrix. Furthermore, HME-processed MUL formulations had an enhanced water solubility and a cumulatively higher release rate of ATCs. The kinetic behavior of the novel system should be further conducted to investigate the prolonged release and stability of ATCs.

## Figures and Tables

**Table 1:** The total flavonoid content, the total phenol content, and the total ATC content of MUL extract.

Sample		Total Flavonoid Contents (mg/100 g)	Total Phenol Contents (mg/100 g)	Total Anthocyanin Contents (mg/100 g)
EtOH ^(7)^	MUL ^(1)^	547.48 ± 14.71 ^a^^*^	1109.77 ± 79.43 ^b^	247.87 ± 8.34 ^b^
Water ^(8)^	MUL	46.47 ± 10.83 ^f^	813.91 ± 10.55 ^f^	95.60 ± 3.33 ^f^
	MUL-CA ^(^^2)^	52.81 ± 9.96 ^c^	2225.28 ± 50.51 ^e^	264.63 ± 6.28 ^d^
	MUL-CA-ALG ^(^^3)^	61.95 ± 39.00 ^d^	1489.78 ± 101.98 ^d^	149.60 ± 5.00 ^e^
	MUL-ESFs ^(^^4)^	114.10 ± 7.60 ^e^	3380.27 ± 53.47 ^g^	259.73 ± 6.71 ^g^
	MUL-ESFs-CA ^(^^5)^	79.20 ± 4.45 ^a^	2668.72 ± 25.35 ^a^	331.08 ± 10.15 ^a^
	MUL-ESFs-CA-ALG ^(^^6)^	68.59 ± 1.20 ^b^	3076.80 ± 37.10 ^c^	308.59 ± 8.08 ^c^

^(1)^ MUL; MUL powder, ^(2)^ MUL-CA; MUL treated with 0.5 M citric acid, ^(^^3)^ MUL-CA-ALG; MUL treated with 0.5 M citric acid and 0.3% sodium alginate, ^(^^4)^ MUL-ESFs; solid formulations of MUL-extrudate, ^(^^5)^ MUL-ESFs-CA; solid formulations of the extrudate of MUL treated with 0.5 M citric acid, ^(^^6)^ MUL-ESFs-CA-ALG; solid formulations of the extrudate of MUL treated with 0.5 M citric acid and 0.3% sodium alginate, ^(^^7)^ EtOH; extraction with 80% ethanol, ^(^^8)^ Water; extraction with water. All the data were expressed as means ± SD (n = 3). * All the values were reported as means ± SD (n = 3). * Means with different letters (a–g) in the same column are significantly different at *p* < 0.05, which is confirmed by performing one-way ANOVA test.

**Table 2:** Color difference meter measuring the color of MUL extracts (25,000 ppm).

Sample		Color	L*, a*, b*
EtOH ^(7)^	MUL	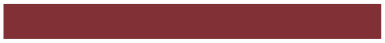	L*; 31.12, a*; 37.50, b*; 17.50
Water ^(8)^	MUL ^(1)^	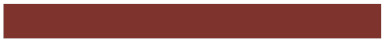	L*; 30.64, a*; 34.67, b*; 22.53
	MUL-CA ^(2)^	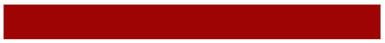	L*; 32.73, a*; 56.64, b*; 48.48
	MUL-CA-ALG ^(3)^	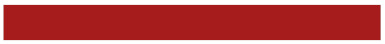	L*; 35.67, a*; 56.03, b*; 41.24
	MUL-ESFs ^(4)^	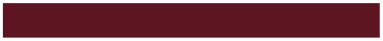	L*; 18.46, a*; 35.20, b*; 14.64
	MUL-ESFs-CA ^(5)^	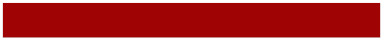	L*; 32.87, a*; 57.17, b*; 48.08
	MUL-ESFs-CA-ALG ^(6)^	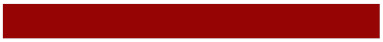	L*; 29.90, a*; 57.25, b*; 48.64

^(1)^ MUL; MUL powder, ^(2)^ MUL-CA; MUL treated with 0.5 M citric acid, ^(3)^ MUL-CA-ALG; MUL treated with 0.5 M citric acid and 0.3% sodium alginate, ^(4)^ MUL-ESFs; solid formulations of the extrudate of MUL, ^(5)^ MUL-ESFs-CA; solid formulations of the extrudate of MUL treated with 0.5 M citric acid, ^(6)^ MUL-ESFs-CA-ALG; solid formulations of the extrudate of MUL treated with 0.5 M citric acid and 0.3% sodium alginate, ^(7)^ EtOH; extraction with 80% ethanol, ^(8)^ Water; extraction with water. L*; defines lightness, a*; denotes the red/green value, b*; denotes the yellow/blue value.

**Table 3:** Water solubility analysis by rheometer and hot-melt-extrusion of MUL ultrafine powder of different chemical formulations.

Formulation	Solubility (%)
MUL ^(1)^	13.26 ± 0.41% ^d^^*^
MUL-CA ^(^^2)^	13.63 ± 0.18% ^cd^
MUL-CA-ALG ^(^^3)^	13.41 ± 0.25% ^d^
MUL-ESFs ^(^^4)^	13.96 ± 0.33% ^c^
MUL-ESFs-CA ^(^^5)^	16.08 ± 0.21% ^b^
MUL-ESFs-CA-ALG ^(^^6)^	17.03 ± 0.32% ^a^

^(1)^ MUL; MUL powder, ^(2)^ MUL-CA; MUL treated with 0.5 M citric acid, ^(3)^ MUL-CA-ALG; MUL treated with 0.5 M citric acid and 0.3% sodium alginate, ^(4)^ MUL-ESFs; only solid formulations of the extrudate of MUL, ^(5)^ MUL-ESFs-CA; solid formulations of the extrudate of MUL treated with 0.5 M citric acid, ^(6)^ MUL-ESFs-CA-ALG; solid formulations of the extrudate of MUL treated with 0.5 M citric acid and 0.3% sodium alginate. * Means with different letters (a–g) in the same column are significantly different at *p* < 0.05, which is confirmed by performing one-way ANOVA test.

**Table 4:** The conditions of HPLC analysis of C3G and C3R.

HPLC System		
Column	YMC-Triart C18	
Flow rate	1.0 mL/min	
Injection volume	10 μL	
Oven temperature	30 °C	
Detector wavelength	535 nm	
Mobile phase	Gradient	
Solvent A	Water:formic acid (90:10)	
Solvent B	Acetonitrile:methanol:water:formic acid (22.5:22.5:40:10)	
Elution time (min)	A	B
0	93	7
35	75	25
45	35	65
46	0	100
50	35	65
60	75	25
70	93	7
75	93	7

## Data Availability

Not applicable.
